# Unusual presentation of a foreign body in the palate

**DOI:** 10.4103/0970-0358.41126

**Published:** 2008

**Authors:** A. Gopalakrishna, T. V. Pavan Kumar

**Affiliations:** Department of Plastic Surgery, Deccan College of Medical Sciences, Hyderabad, AP, India

Dear Sir,

We would like to present a case of a foreign body embedded in the palate which was misdiagnosed as a cleft/fistula in the palate. The problem in diagnosis was because of the difficulty in examining the child without anesthesia, the uncooperative attitude of the parents and the clinicians’ disregard for the history given by the mother.

Recently a eleven month-old child was referred to the Department of Plastic Surgery in our hospital by a general practitioner with the diagnosis of a cleft palate. The child was uncooperative and would not allow examination of the oral cavity. The parents were equally uncooperative. Although there was no history of food or milk escaping from the nose, there appeared to be a tissue defect in the hard palate appearing as an irregularly shaped black recess in the mid-line. The soft palate could not be visualized in the routine examination of the child. The mother had noticed this phenomenon at the age of one month and it was assumed by everybody that she had missed the finding at birth and a diagnosis of cleft palate was made by the general practitioner and pediatrician treating the patient.

**Figure 1:**
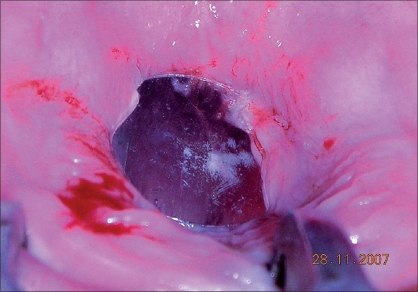
Foreign body lodged in hard palate

**Figure 2:**
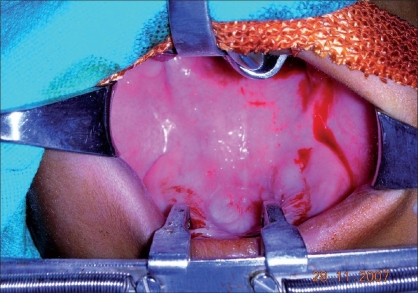
Intact palate after removal of foreign body

**Figure 3:**
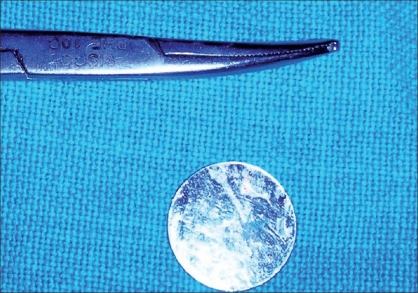
Metallic foreign body removed from hard palate

On subjecting the child to examination under anesthesia, a shining plastic disc of the kind used in garment manufacture was found firmly adherent to the hard palate [Figures 1-3] in the mid-line with some palatal tissue overlapping the edge of the disc. On removing the disc, the palate was found to be normal and intact.

This reinforces the old lesson that history-taking is of prime importance in clinical practice.

